# Association between surgical tracheostomy and chronic tracheal stenosis: A retrospective, single-center study

**DOI:** 10.3389/fmed.2022.1050784

**Published:** 2022-12-05

**Authors:** Yuki Kuwabara, Kentaro Yamakawa, Seiko Okui, Erica Miyazaki, Shoichi Uezono

**Affiliations:** Department of Anesthesiology, Jikei University School of Medicine, Tokyo, Japan

**Keywords:** tracheal stenosis, surgical tracheostomy, triangulation, intubation, computed tomography

## Abstract

**Background:**

Tracheal stenosis is a major complication of tracheostomy. Accordingly, anesthesiologists tend to select a smaller endotracheal tube (ETT) than usual for patients with a prior tracheostomy history, regardless of the presence or absence of respiratory symptoms. However, it likely comes from our trial and error, not scientific evidence. Therefore, in this study, we retrospectively examined the association between traditional surgical tracheostomy and tracheal stenosis as assessed by transverse computed tomography (CT).

**Methods:**

Patients who underwent surgery for head and neck cancer from January 2010 to December 2013, with a temporary tracheostomy closed within a couple of months, were included. Exclusion criteria were tracheostoma before surgery, permanent tracheostomy, or insufficient CT follow-up. Transverse CT slices were measured 2 cm above and below the tracheostomy site (0.5 cm/slice for a total of 9 slices). The minimum cross-sectional tracheal area and horizontal and vertical diameters in transverse CT slices were compared before (baseline: BL), 6 months (6M) and 12 months (12M) after tracheostomy. Tracheal stenosis was defined as a decrease in the minimum cross-sectional tracheal area compared to BL.

**Results:**

Of 112 patients, 77 were included. The minimum tracheal area was significantly decreased at 6M and 12M compared to BL (BL: mean 285 [SD 68] mm^2^, 6M: 267 [70] mm^2^, *P* < 0.01 vs. BL, 12M: 269 [68] mm^2^, *P* < 0.01 vs. BL), and the localization was predominantly at or above the tracheostomy site at 6M and 12M. Tracheal stenosis was identified in 55 patients at 6M and in 49 patients at 12M without any respiratory symptoms. With regard to horizontal and vertical diameter, only horizontal diameter was significantly decreased at 6M and 12M compared to BL (BL: 16.8 [2.4] mm, 6M: 15.4 [2.7] mm, *P* < 0.01 vs. BL, 12M: 15.6 [2.8] mm, *P* < 0.01 vs. BL).

**Conclusion:**

Conventional surgical tracheostomy was associated with a decreased horizontal diameter of the trachea. It resulted in a decreased cross-sectional tracheal area in more than one-half of the patients; however, no patient complained of any respiratory symptoms. Therefore, even without respiratory symptoms, prior tracheostomy causes an increased risk of tracheal stenosis, and using a smaller ETT than usual could be reasonable.

## Introduction

Tracheostomy is commonly performed in cases of upper airway obstruction and for patients who require prolonged mechanical ventilation (1). The benefits of tracheostomy include upper airway management, as well as decreased risk of ventilator-associated pneumonia in patients intubated long-term (2, 3). However, a tracheostomy can alter the tracheal shape around the surgical site to result in an A-frame or triangular-shaped deformity (4–6), which can lead to tracheal stenosis (7). Tracheal stenosis of > 30% to 50% of the original size is thought to cause respiratory symptoms, and the incidence of symptomatic tracheal stenosis after tracheostomy is reported to be 1 to 6% (7–9). On the other hand, asymptomatic tracheal stenosis after tracheostomy has not been well examined (10, 11), since mild tracheal stenosis is not often subject to active treatment. Regardless of the respiratory symptom, anesthesiologists tend to select a smaller endotracheal tube (ETT) than usual in general anesthesia for patients with a prior tracheostomy history without distinct scientific evidence.

At our institute, otolaryngologists perform a surgical tracheostomy to prevent postoperative upper airway obstruction during surgery for head and neck tumorectomy and flap reconstruction. The tracheostomy is usually closed for about a month once respiratory safety is confirmed. And their cancer follow-up is performed with imaging over time, including trachea around tracheostomy. Some of these patients without any respiratory symptoms may visit an operation room again to undergo a different kind of surgery. In such cases, the choice of ETT is sometimes discussed.

Therefore, the purpose of the present study was to retrospectively examine the association of conventional surgical tracheostomy with asymptomatic tracheal stenosis over time as measured by transverse computed tomography (CT) in head and neck cancer patients.

## Materials and methods

This retrospective study was approved by the Jikei University Certified Review Board, which provided a waiver of written informed consent (number 29-127[8743]). This study was conducted in accordance with the principles of the Helsinki Declarations.

Patients who underwent head and neck cancer surgery from January 2010 to December 2013, with a temporary tracheostomy closed within a couple of months, were included in the study. Exclusion criteria were tracheostoma before surgery, permanent tracheostomy, or insufficient CT follow-up. Patients were performed a tracheostomy with a U-shaped tracheal incision under general anesthesia, and ventilated through the tube in the tracheostoma during the surgery. After respiratory safety was confirmed, the tracheostomy tube was removed to close the site.

### Data collection and tracheal measurement

**FIGURE 1:**
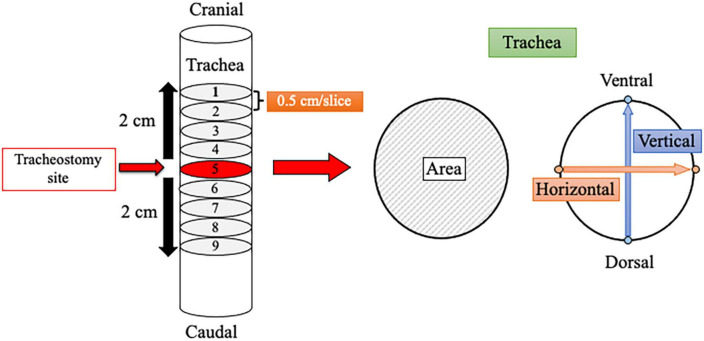
Schematic of a method for measuring the tracheal lumen using computed tomography. Measurement was performed over a distance of 4 cm above and below the tracheostomy site. Each image slice was set to 0.5 cm, and 9 slices were analyzed. The minimum tracheal area among the 9 slices was selected for each time point (baseline, 6 months, and 12 months). Horizontal and vertical tracheal diameters were also measured.

Demographic information, including age, sex, height, body weight, body mass index, and medical history, was reviewed and collected from medical records. All patients underwent CT before (baseline [BL]) and after surgery at 6 months (6M) and 12 months (12M) for cancer evaluation. The presence and extent of tracheal stenosis were analyzed retrospectively using the CT images, which were set to 0.5 cm per slice. In the transverse plane, cross-sectional tracheal area and vertical and horizontal diameters were measured for each of the 9 slices over a distance of 4 cm (2 cm above and below the tracheostomy site). The cross-sectional tracheal area was calculated by tracing around the trachea, and diameters were measured by determining 2 sites of the tracheal wall in each of the horizontal and vertical directions (Figure 1) with picture archiving and communication system image display (Synapse; Fujifilm Corp., Tokyo, Japan). The minimum cross-sectional area for each patient at each time point (BL, 6M, and 12M) was used for analysis. Tracheal stenosis was defined as a decrease of the minimum cross-sectional area at 6M or 12M compared to BL.

### Statistical analysis

Statistical analysis and figure creation were performed with GraphPad Prism software (version 8, GraphPad Software Inc., San Diego, CA) and JMP Pro 16.0.0 (512340) (SAS Institute., Cary, NC, USA). Statistical normality was confirmed with the Shapiro-Wilk test. Quantitative data are presented either as mean [SD] or median [first, third quartile], per statistical normality, and qualitative data are presented as *n* (%). A one-way repeated-measures analysis of variance with a Tukey *post hoc* test or Friedman test with a Dunn *post hoc* test was performed to test tracheal measurements per statistical normality. A *P* value <0.05 was considered statistically significant.

## Result

**FIGURE 2:**
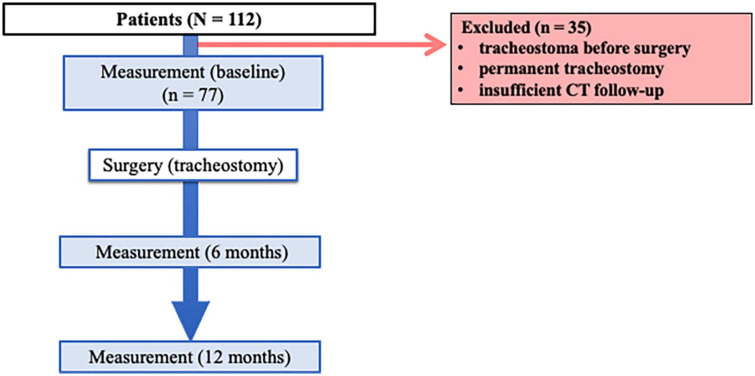
Flow of study protocol. Of 112 patients who underwent surgery for neck and head cancer, 77 were included in the study. Computed tomography was performed before surgery (baseline) and at 6 and 12 months after surgery.

A total of 112 patients who underwent surgery and tracheostomy were identified, and 77 were deemed eligible for analysis (Figure 2). Patient demographic characteristics are summarized in Table 1. The average age was 62 (11) years, and 57 (74%) patients were male. A total of 55 (71%) patients were smokers. The average time to tracheostomy closure was 34 (14) days.

**TABLE 1:** Patient demographic characteristics (*n* = 77).

Age, y, mean (SD)	62 (11)
Sex (male/female), *n* (%)	57 (74)/20 (26)
Height, cm, mean (SD)	163.4 (7.9)
Body weight, kg, mean (SD)	58.8 (11.3)
Body mass index, kg/m^2^, mean (SD)	21.9 (3.3)
Hypertension, *n* (%)	34 (44.2)
Diabetes, *n* (%)	12 (15.6)
COPD, *n* (%)	8 (10.4)
Smoker, *n* (%)	55 (71.4)

COPD, chronic obstructive pulmonary disease.

No patients had any respiratory symptoms.

**FIGURE 3:**
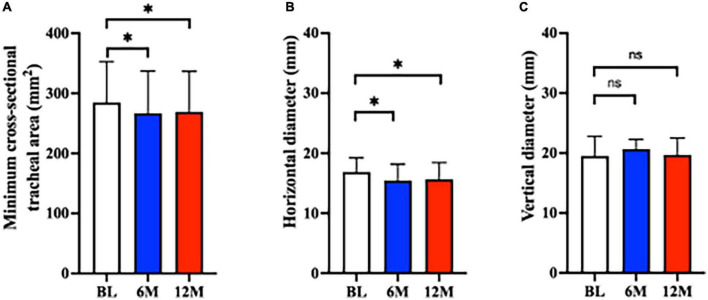
Tracheal stenosis after tracheostomy. The minimum tracheal area decreased significantly at 6M and 12M (both *P* < 0.01 vs. BL). There was no difference between 6M and 12M (*P* = 0.66). *P < 0.01. (B) The horizontal tracheal diameter decreased significantly at 6M and 12M compared to BL (both *P* < 0.01 vs. BL). There was no difference between 6M and 12M (*P* = 0.43). *P < 0.01. (C) The vertical diameter did not change over time (6M: *P* = 0.23 vs. BL; 12M: *P* = 0.32 vs. BL). No change was observed between 6M and 12M (*P* > 0.99). Bar graphs are shown as mean ± SD (A,B) and median, first, and third quartile (C). 6M: 6 months after surgery, 12M: 12 months after surgery, BL: baseline, ns, not significant.

The minimum cross-sectional tracheal area was significantly decreased at 6M and 12M after tracheostomy compared to BL (BL: 285 [68] mm^2^; 6M: 267 [70] mm^2^, *P* < 0.01 vs. BL; 12M: 269 [68] mm^2^, *P* < 0.01 vs. BL) (Figure 3A). Of the 77 analyzed patients, tracheal stenosis was observed in 55 (71.4%) patients by 11.9% (9.4%) at 6M, and in 49 (63.6%) patients by 12.2% (8.8%) at 12M.

**FIGURE 4:**
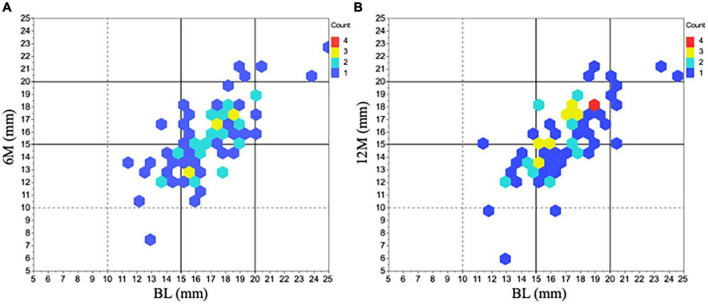
Horizontal diameter changes in minimum tracheal area before and after tracheostomy. The distribution of horizontal diameter changes in all 77 patients was represented at 6M (A) and 12M (B) compared to BL. The most predominant range in the initial horizontal diameter was within 15 ∼ 20 mm at BL (*n* = 56). After tracheostomy, many of the diameters shifted shorter than the initial one. 6M: 6 months after surgery, 12M: 12 months after surgery, BL: baseline.

With respect to horizontal and vertical tracheal diameters, the horizontal diameter was significantly decreased compared to BL (BL: 16.8 [2.4] mm, 6M: 15.4 [2.7] mm, *P* < 0.01 vs. BL, 12M: 15.6 [2.8] mm, *P* < 0.01 vs. BL) (Figure 3B). The change in the horizontal diameter compared to BL was –8.1% [12%] at 6M and –7.1% [12%] at 12M. Figure 4 demonstrates the horizontal diameter distribution in each patient’s minimum cross-sectional area at BL, 6M (Figure 4A) and 12M (Figure 4B).

No significant difference was observed for vertical diameter (BL: 19.5 [17.2, 22.8] mm, 6M: 20.7 [17.6, 22.3] mm, *P* = 0.23 vs. BL, 12M: 19.7 [17.5, 22.5] mm, *P* = 0.32 vs. BL) (Figure 3C).

**FIGURE 5:**
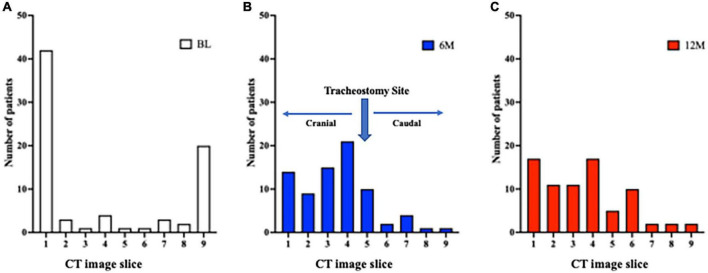
Localization of the site of minimum tracheal area before and after tracheostomy. (A) A total of 9 CT slices were obtained for each patient (cranial to caudal, with tracheostomy site in the middle [slice 5]). At BL, the site of minimum tracheal area was at the most cranial (slice 1) or most caudal (slice 9) slice. (B,C) At 6M and 12M, the site of minimum tracheal area was predominantly at or above the tracheostomy site. 6M: 6 months after surgery, 12M: 12 months after surgery, BL: baseline, CT: computed tomography.

**FIGURE 6:**
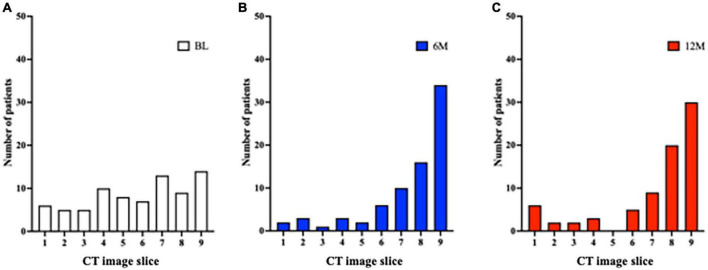
Localization of the site of maximum tracheal area before and after tracheostomy. (A) A total of 9 CT slices were obtained for each patient (cranial to caudal, with tracheostomy site in the middle [slice 5]). At BL, there was no predominant localization of the site of maximum tracheal area. (B,C) At 6M and 12M, the site of maximum tracheal area was localized below the tracheostomy site (caudally). 6M: 6 months after surgery, 12M: 12 months after surgery, BL: baseline, CT: computed tomography.

The minimum cross-sectional area for the 9 CT slices for each patient was localized to the most caudal or cranial level at BL (Figure 5A). It was predominantly at or above the tracheostomy site at 6M and 12M (Figures 5B,C). Conversely, the localization of the maximal tracheal area was predominantly below the tracheostomy site (caudally) at 6M and 12M (Figures 6B,C), whereas no predominant localization was observed at BL (Figure 6A).

## Discussion

**FIGURE 7:**
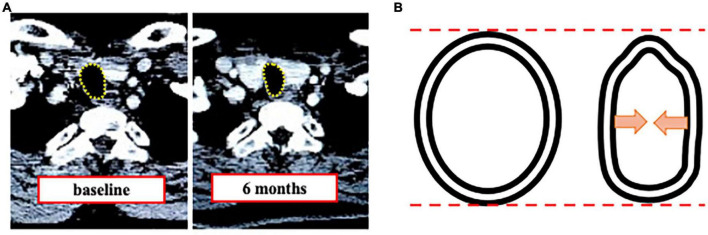
Tracheal stenosis after tracheostomy. (A) Representative computed tomography image of tracheal stenosis. (B) Schematic showing triangulation after tracheostomy.

We measured tracheal area and diameter in CT to assess tracheal stenosis by tracheostomy and demonstrated decreased tracheal area and horizontal diameter. Our results showed that conventional surgical tracheostomy was associated with (1) a decrease of the minimum cross-sectional tracheal area in more than one-half of patients at 6M (11.9% area reduction), which was maintained at 12M (12.2% area reduction) without any respiratory symptom, (2) localization of the minimum cross-sectional area in the cranial direction and the maximum tracheal area in the caudal direction from the tracheostomy site, and (3) a significantly decreased horizontal tracheal diameter at 6M and 12M compared to BL without changes in vertical diameter. In addition, the alteration causes a trachea for triangular-shaped stenosis after tracheostomy (Figure 7).

In most reports regarding tracheostomy, bronchoscopy has been used to assess for tracheal stenosis (12). However, detailed changes in tracheal diameter by numerical value cannot be measured by bronchoscopy, and it is relatively invasive compared to CT (13–16). Thus, this is the first study to show detailed tracheal measurements over time in patients with asymptomatic tracheal stenosis after conventional surgical tracheostomy.

## Tracheal stenosis – decreased area after tracheostomy

The incidence and degree of chronic tracheal stenosis after surgical tracheostomy of the intact trachea, which closes for about a month, especially how the tracheal lumen changes during follow-up, has not been fully elucidated. James et al. studied tracheal stenosis after surgical tracheostomy in maxillofacial surgery and reported 8.8% without any respiratory symptoms (11). In that study, they measured the shortest anteroposterior or transverse tracheal diameter with CT or magnetic resonance imaging. They demonstrated the tracheal stenosis as the change in diameter, not a numerical value. We speculate that the different incidences between the two studies were the different definitions of tracheal stenosis and the various duration of the cannulation period. We defined tracheal stenosis as a decrease in the minimum cross-sectional tracheal area compared to BL, because our focus was on overall tracheal deformity, including the examination of horizontal and vertical diameters. Furthermore, the longer intubation period in the present study (34 [13] days) might have increased the number of patients with tracheal stenosis (7, 11, 17).

Also, concerning the comparison to percutaneous dilatational tracheostomy (PDT), PDT has been widely accepted as an alternative surgical tracheostomy and is considered a safe strategy (18–20). An observational study demonstrated that 15 (31%) of 48 long-term PDT patients (average 30 months observation) developed tracheal stenosis more than 10% in diameter by CT measurement. Furthermore, one (2%) patient had severe stenosis, a greater than 50% reduction from the original trachea (21). The present study showed that 27 (35%) patients decreased tracheal area by more than 10% from the original trachea at 12M, and one patient decreased by more than 50% at 6M, followed by 30% at 12M. The definitions of tracheal stenosis were different (tracheal diameter narrowing vs. tracheal area reduction), but surgical tracheostomy can be comparable with PDT from the point of tracheal stenosis after tracheostomy.

In the localization of tracheal stenosis, the minimum tracheal area was seen at the proximal and distal locations at BL, where there might be difficulty with intubation, even in patients without stenosis. After tracheostomy, the minimum tracheal area was predominantly observed above the tracheostomy site, consistent with prior reports examining symptomatic tracheal stenosis or deformity by endoscopy (6, 12). On the other hand, the maximum area was broadly distributed across the trachea at BL. Interestingly, we found that the maximum tracheal area was localized in the caudal direction when the tracheostomy tube was placed for approximately a month until respiratory safety was confirmed; this might prevent the development of granulation tissue and resulting stenosis.

### Tracheal stenosis – decreased horizontal diameter after tracheostomy

In the present study, the horizontal diameter decreased by 1.2 to 1.4 mm from BL over time, but the vertical diameter did not change compared to BL. This heterogeneous change caused tracheal deformity, leading to tracheal stenosis (Figure 7) (4, 22). Although it has been reported that tracheostomy can cause tracheal stenosis due to the alteration of the trachea to be a triangular shape (14), there have been no prior studies on how much each diameter changes after conventional surgical tracheostomy for the horizontal and vertical direction. The evidence of the decreased horizontal diameter gives anesthesiologists useful information to select an ETT.

### Tracheal stenosis and endotracheal tube selection

Patients with a triangular-shaped trachea can be difficult to intubate (5). As shown in Figure 4, the horizontal diameter of many patients in the present study was 15.0 ∼ 19.9 mm at BL. Then after tracheostomy, the horizontal diameter decreased to 10.0 ∼ 14.9 mm in almost half of the study population. Of note, the decrease of the horizontal diameter after tracheostomy was 1.4 [1.9] mm at 6M and 1.2 [1.9] mm at 12M, which was very variable. Comparing various types of available ETTs (Table 2), all with an inner diameter of ≥7.5 mm have an outer diameter of > 10.0 mm. In addition, it is important to consider the cuff when selecting the ETT because it could get stuck if the outer diameter is close to the shortest horizontal diameter in the trachea. These facts show us that downsizing ETT might be reasonable for a patient with a short horizontal diameter of less than 14.9 mm before tracheostomy to prevent airway trouble, regardless of respiratory symptoms.

**TABLE 2:** Endotracheal tube diameter measurements.

Manufacturer	Inner diameter (mm)	Outer diameter (mm)
Covidien, Hi-Lo	8.0	10.8
	7.5	10.2
	7.0	9.5
	6.5	8.9
	6.0	8.2
Teleflex, Hi-Lo	8.0	11.4
	7.5	10.9
	7.0	10.4
	6.5	9.9
	6.0	9.4
Smiths Medical, Hi-Lo	8.0	10.9
	7.5	10.3
	7.0	9.6
	6.5	8.9
	6.0	8.2

We suggest that anesthesiologists evaluate neck CT images if available to ensure an appropriate ETT size with the right cuff volume is selected before intubation for patients with a history of surgical tracheostomy because of variable changes in tracheal diameter. We believe that the present study provides anesthesiologists with practical information regarding the right selection of ETTs with asymptomatic tracheal stenosis after tracheostomy and the usefulness of neck CT for preoperative evaluation.

## Limitations

This study has a few limitations. First, this was a retrospective study, and causality was not assessed. Second, several reports indicate that cuff pressure is a risk factor for tracheal stenosis (9, 23); however, all of the patients in this study underwent tracheostomy during surgery, and cuff pressure was monitored right after intubation. Third, we analyzed a total distance of 4 cm (2 cm above and below the tracheostomy site) by transverse-plane CT, not the entire trachea (24). However, we focused on the relationship between tracheostomy and tracheal stenosis. We believe that assessment 2 cm above and below the tracheostomy site is enough to evaluate for tracheal stenosis before surgery.

## Conclusion

The present CT study detailed the development of asymptomatic tracheal stenosis after tracheostomy. Even in patients without respiratory symptoms, the decreased cross-sectional tracheal area at 6M and 12M after tracheostomy appeared to be due to a decrease in horizontal tracheal diameter. The use of CT can help to evaluate tracheal stenosis before anesthesia in patients with prior tracheostomy.

## Data availability statement

The raw data supporting the conclusions of this article will be made available by the authors, without undue reservation.

## Ethics statement

The studies involving human participants were reviewed and approved by the Jikei University. Written informed consent for participation was not required for this study in accordance with the national legislation and the institutional requirements.

## Author contributions

YK and KY contributed to the study’s conception and design and wrote the manuscript. SO, EM, YK, and KY performed the data collection and analysis. SU supervised this study. All authors have reviewed and approved the final manuscript.
